# A_3_ Adenosine and P2X7 Purinergic Receptors as New Targets for an Innovative Pharmacological Therapy of Malignant Pleural Mesothelioma

**DOI:** 10.3389/fonc.2021.679285

**Published:** 2021-10-01

**Authors:** Fabrizio Vincenzi, John Charles Rotondo, Silvia Pasquini, Francesco Di Virgilio, Katia Varani, Mauro Tognon

**Affiliations:** ^1^ Department of Translational Medicine, Pharmacology Section, University of Ferrara, Ferrara, Italy; ^2^ Department of Medical Sciences, Experimental Medicine Section, Laboratories of Cell Biology and Molecular Genetics, University of Ferrara, Ferrara, Italy; ^3^ Department of Medical Sciences, Experimental Medicine Section, Pathology Unit, University of Ferrara, Ferrara, Italy

**Keywords:** malignant pleural mesothelioma, A_3_ adenosine receptor, P2X7 receptor, Cl-IB-MECA, AZ10606120, anticancer therapy

## Abstract

Human malignant pleural mesothelioma (MPM) is a rare, but aggressive tumor of the serosal cavities whose 5-year survival rate is 15%. At present, there are no effective therapies for MPM. Although recent findings suggest that A_3_ adenosine (A_3_AR) and P2X7 (P2X7R) receptors can be employed as antitumoral pharmacological targets in MPM, their potential role in a combined therapy is currently unknown. The A_3_AR agonist Cl-IB-MECA and the P2X7 receptor antagonist AZ10606120, as a single compound or in combination, were investigated *in vitro* for their anti-tumor activities. Assays were carried out in MPM cell lines IST-Mes2 and MPP89 and in primary human normal mesothelial cells (HMCs), as control. Single treatment with Cl-IB-MECA reduced cell proliferation and favored a pro-apoptotic effect in both MPP89 and IST-Mes2 cell lines, whereas AZ10606120 inhibited cell proliferation and induced apoptosis in IST-Mes2, only. The combined treatment with Cl-IB-MECA and AZ10606120 reduced cell proliferation and favored apoptosis in MPP89 and IST-Mes2 cell lines, whereas no synergistic effect was detected. These data cumulatively suggest the absence of a synergistic effect in combined targeting of A_3_ adenosine and P2X7 receptors of MPM cell lines. This study may stimulate further investigations aimed at determining new combinations of antitumor compounds and more effective therapeutic strategies against MPM.

## Introduction

Human malignant pleural mesothelioma (MPM) is a highly aggressive tumor of the serosal cavities with a 5-year survival rate of approximately 15%, whereas MPM patients die approximately one year after diagnosis. Although MPM is considered a rare tumor, statistical data estimate that one-quarter million people could die of this malignancy in Europe in the next three decades (1). Its incidence is increasing significantly, being cause of about 40,000 deaths each year worldwide (2).

MPM exhibits a strong association to asbestos fiber exposure, such as crocidolite, amosite, and chrysotile (3–5). As for other tumors (6–8), the genetic background/mutations in different genes is another important risk factor of the MPM onset. Indeed, genetic factors, including truncated mutants in the tumor suppressor *BRCA1 associated protein-1 (BAP1)* gene are involved in the development of this tumor (9). Notably, it has been reported that MPM tumorigenesis is due to the combination of the host’s genetic and environmental exposure to asbestos fibers (10–12). Epidemiological data suggest that the genetic background is essential in determining the individual susceptibility to the asbestos-related MPM onset (11). In fact, only a fraction of subjects carrying a specific genetic background develop this tumor (13), which is estimated to be approximately 1%-10% of ex-exposed asbestos workers (14–16), depending on the study being considered (2).

Additional factors, such as radiations and the Simian Virus 40 (SV40) infection (17), have been considered as possible agents responsible for the MPM onset (18). Particularly, previous *in vitro* experiments indicate that the exposition of pleural mesothelial cells with γ-rays and asbestos fibers may provoke oncogenic effects such as cell cycle control alterations (18). Different investigations have been reported the detection of SV40 footprints, at a higher prevalence, in MPM specimens, as well as anti-SV40 IgG antibodies in sera from MPM patients (17). Other studies reported negative data (17).

At present, there are no effective therapies for MPM (5). Data from our laboratories indicate that the A_3_ adenosine receptor (A_3_AR) can be potentially employed as a target for a MPM pharmacological therapy (19, 20). Indeed, treatments with A_3_AR agonist 2-chloro-N6-(3-iodobenzyl) adenosine-5’-N-methyl-uronamide (Cl-IB-MECA, also referred to as CF102) induce MPM cell growth inhibition (19, 20). A_3_AR agonists have been assayed, with a good clinical efficacy, in different malignancies such as breast cancer (21), melanoma (22) and pancreatic cancer (23, 24). The potential role of Cl-IB-MECA has been reported by many studies (20, 25–27). For instance, the anti-neoplastic effect of this A_3_AR agonist has been reported in human lung (28), prostate (29) and breast cancer cells (30, 31), in human melanoma cells (32) as well as in murine lymphoma cells (33).

Another potential target for pharmacological MPM therapy is represented by the purinergic receptor P2X7R (34). This receptor is expressed in MPM cells, while being completely absent in normal human mesothelial cells (HMC). *In vitro* experiments indicated that targeting P2X7R with the antagonist AZ10606120 induces a strong inhibition of MPM cell growth, in association with the release of the cytoplasmic marker lactate dehydrogenase (LDH) (34).

*In vivo*, the systemic administration of AZ10606120 remarkably reduces tumor growth in immunocompromised, *nude/nude* mice, underwent either subcutaneous (s.c.) or intraperitoneal (i.p.) inoculation of MPM cells (34). Similarly, other *in vitro/in vivo* studies focused on neuroblastoma and pancreatic cancers reported that P2X7R can be employed as a target for anticancer therapy (23, 24, 34, 35).

Although the antitumor effects of both A_3_AR agonist Cl-IB-MECA and P2X7R antagonist AZ10606120 on MPM have been reported, their potential role in a combined therapy is currently unknown.

The present study was carried out to investigate whether Cl-IB-MECA and AZ10606120 assayed in MPM treated cells enhance the MPM cell growth arrest when simultaneously administered. The combined treatment of the A_3_AR agonist Cl-IB-MECA and P2X7R antagonist AZ10606120 was investigated in order to verify whether they reduce the cell proliferation and enhance the pro-apoptotic effect in MPM cell lines *in vitro*.

## Methods

### Compounds

The A_3_AR agonist 2-chloro-N6-(3-iodobenzyl) adenosine-5’-N-methyl-uronamide (Cl-IB-MECA) and the P2X7R inhibitor AZ10606120 (Tocris Bioscience, Ellisville, MS, USA) (Tocris Bioscience, Ellisville, MS, USA) were employed as a single compound or together for *in vitro* treatments (19, 34). The P2X7R inhibitor AZ10606120 and the A_3_AR agonist Cl-IB-MECA, as stock solutions, were dissolved in DMSO at a concentration of 25 mM and 100 mM, respectively.

### Cell Cultures and Treatments

MPM cell lines, MPP89 and IST-Mes2, as well as HMCs were cultured in DMEM F-12 medium supplemented with 10% FBS and maintained at 37°C in a 5% CO_2_-humidified atmosphere. MPM cells and primary HMCs were processed as previously described (34). Cells were plated in 96 well plates and cultured overnight in serum-free DMEM-F12. MPM and HMC cells were treated with different concentrations of Cl-IB-MECA (1 nM - 1 µM) in the absence or in the presence of AZ10606120 (300 nM) for 24-48-72 hours (19, 34). Cisplatin (100 µM) and Etoposide (30 µM) drugs were used as positive controls during the proliferation and apoptosis assays, respectively.

### A_3_AR Saturation Binding Experiments

Saturation binding experiments at A_3_AR were performed using [^3^H]-5N-(4-methoxyphenyl-carbamoyl) amino-8-propyl-2-(2-furyl) pyrazolo-[4,3-e]-1,2,4-triazolo-[1,5-c]pyrimidine ([3H]-MRE 3008F20, specific activity 67 Ci/mmol; GE Healthcare) as a radioligand (19). Membranes (80 μg of protein/assay) were incubated with [3H]-MRE 3008F20 (0.1–30 nM) at 4°C for 150 min. Nonspecific binding was determined in the presence of MRE 3008F20 (1 μM). At the end of the incubation time, bound and free radioactivity were separated by filtering the assay mixture through Whatman GF/B glass fiber filters (Whatman, Maidstone, UK) by using a Brandel cell harvester (Brandel Instruments, Gaithersburg, MD, USA). The filter-bound radioactivity was counted using a Perkin Elmer 2810 TR liquid scintillation counter (Perkin Elmer).

### Cell Proliferation Assay

Cell proliferation was evaluated by using CyQUANT NF Cell Proliferation Assay Kit (Invitrogen) according to the manufacturer’s instructions (19, 34). This method detects cell proliferation by measuring the cellular DNA content, *via* fluorescent DNA binding dye in combination with a plasma membrane permeabilization reagent. At the end of each treatment, medium was aspirated from wells and replaced with the dye binding solution (composed by CyQUANT^®^ NF and Hank’s balanced salt solution), incubated for 60 minutes at 37°C. Then, the fluorescence intensity was read using Ensight Multimode plate reader (Perkin Elmer) with excitation at 485 nm and emission detection at 530 nm.

### Cell Viability Assay

MTT (3-(4,5-dimethylthiazol-2-yl)-2,5-diphenyl tetrazolium bromide) assay was performed to detect cell viability. At the end of the treatment, MTT solution was added to each well at a final concentration of 0.5 mg/ml. The viable cells contain NAD(P)H-dependent oxidoreductase enzymes which reduce the MTT to formazan. After a 4-hour incubation at 37°C, a formazan solubilization solution composed of HCl acidified isopropanol was added. Following complete solubilization of the purple formazan crystals, the absorbance of the samples was measured at 570 nm with a reference wavelength of 690 nm in an Ensight Multimode plate reader (Perkin Elmer).

### Apoptosis Assay

Cell apoptosis was evaluated by using CellEvent Caspase-3/7 Green Detection Reagent (Invitrogen) according to the manufacturer’s instructions (19, 34). This assay consists of a fluorogenic substrate for activated caspases-3 and -7. After the activation of these caspases in apoptotic cells the substrate is cleaved enabling the dye to bind DNA and producing a fluorogenic reaction. The cells were incubated with the substrate solution for 30 minutes at 37°C, then the fluorescence intensity was read using Ensight Multimode plate reader (Perkin Elmer) with excitation/emission at 502/530 nm.

### Annexin V/SYTOX™ AADvanced Experiments

At the end of each treatment, cells were detached using Accutase (Life Technologies) and subsequently stained with Annexin V Alexa Fluor™ 488 Ready Flow Conjugate to 1 x 10^5^ cells in 100 µl of Annexin Binding Buffer (Life Technologies). Cells were then incubated for 5 minutes at 25°C, followed by the addition of 1 µM SYTOX™ AADvanced™ Dead Cell Stain. Data were acquired on an Attune NxT Flow Cytometer (Thermo-Fisher Scientific, Paisley, UK) equipped with a 488 nm laser for excitation. Fluorescence emission was collected using a 530/30 BP filter and a 695/40 BP filter for Annexin V Alexa Fluor™ 488 and SYTOX™ AADvanced™, respectively (36). Cells were gated according to physical parameters and cell aggregates were removed from the analysis.

### Western Blot Analyses

Cells were plated in 175 cm2 cell culture flask. At 70% of confluence, cells were treated for 72 hours with drugs as previously describe. At the end of incubation, cells were detached and washed with cold PBS. Pellets were lysed with Ripa buffer supplemented with 1mM PMSF, protease inhibitor cocktail, oxophos stop (all by Sigma Aldrich). Protein concentration was measured with Bradford assays. Fifteen µg of protein were loaded onto a NUPAGE BIS-TRIS 4/12% precast gel (life technologies, Monza, Italy) and transferred to nitrocellulose membrane (GE health care-life sciences, Milano Italy). Nonspecific binding site were blocked with 5% skin milk in TBS buffer and nitrocellulose stripes were then incubated overnight with primary antibodies at 4°C. The rabbit anti non-muscle Myosin IIA antibody (Abcam cat n. ab 75590) was diluted 1:3000 in TBS-t Buffer and 2.5% BSA. The rabbit anti cleaved Parp-1Asp214 (Cell signaling #9541) was diluted 1:1000 in TBS-t Buffer and 5% skin milk. The rabbit anti cytochrome C antibody (Abcam cat.n ab133504) was diluted 1:300 in TBS-t Buffer and 5% skin milk. Membranes were incubated with secondary antibody goat antirabbit HRP conjugated antibodies (cat n 31460, Invitrogen, Termofischer Scientific) at 1:3000 dilution for 1 hour at room temperature. ECL reagent was used for detection with LI-COR blot scanner (LI-cor Biosciences, Lincoln, NE, USA). Western blot analysis for cleaved Parp-1Asp214 was performed on total proteins isolated from cells, while cytochrome C levels were evaluated in the subcellular fraction of MPM and HMC cells, as reported (37).

### Statistical Analysis

Statistical analysis of the data was performed by one-way analysis of variance (ANOVA) (38, 39) followed by Bonferroni’s *post hoc* test of n=3 independent experiments conducted in triplicate. Statistical analyses were performed using Graph Pad Prism version 5.0 for Windows (Graph Pad, La Jolla, CA, USA) (40, 41). P-values <0.05 were considered statistically significant (42).

## Results

### A_3_AR Expression in MPM Cell Lines

**Figure 1:**
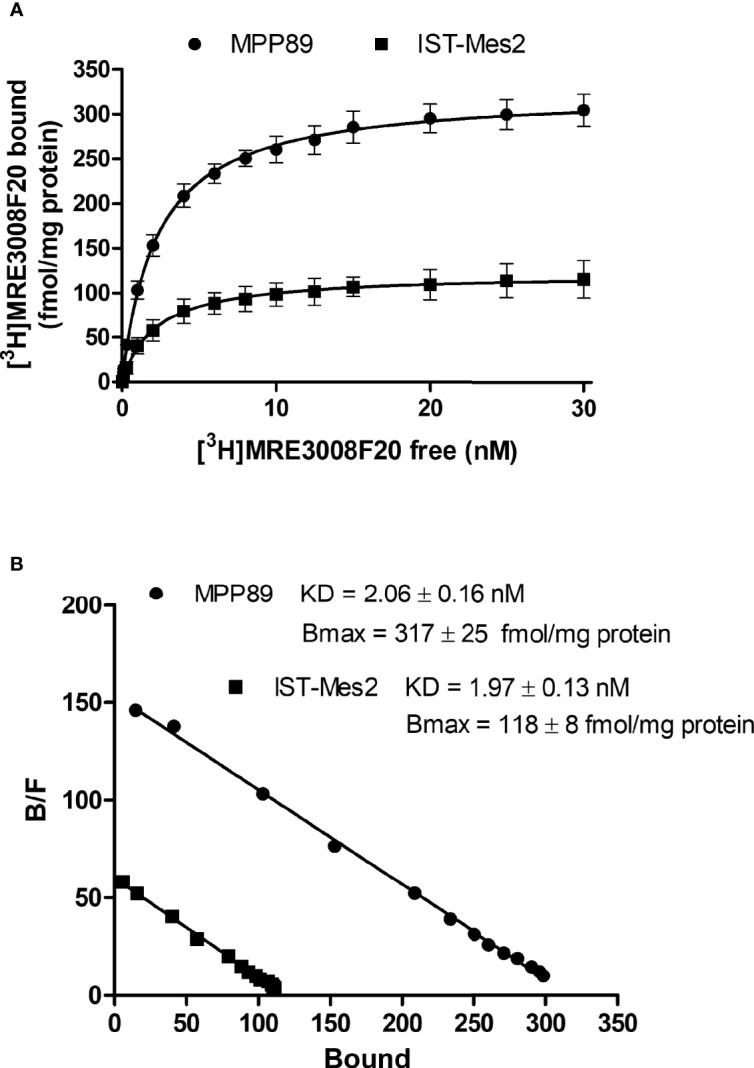
A_3_AR saturation binding experiments. Saturation curves **(A)** and relative Scatchard plots **(B)** of [^3^H]-MRE3008F20 binding on IST-Mes2 and MPP89. Results are represented as mean ± standard error of mean (SEM) of n=3 independent experimental replicates.

Saturation binding experiments performed in MPM cell lines, revealed a lower density of A_3_AR in IST-Mes2 cells in comparison to MMP89 with Bmax values of 118 ± 8 fmol/mg protein and 317 ± 25 fmol/mg protein, respectively (**Figure 1**). The affinity, expressed as K_D_ values, was similar between the two MPM cell lines.

### Determination of the Optimal Concentration of A_3_AR Agonist Cl-IB-MECA in MPM Cells

**Figure 2:**
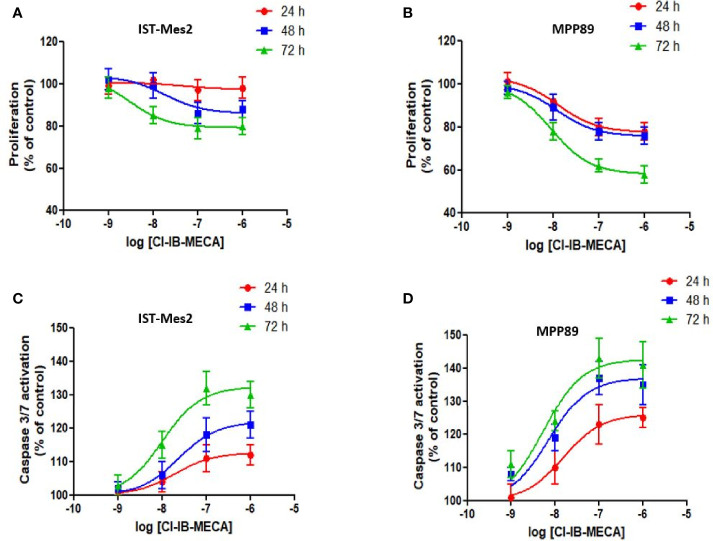
Dose-response assays with Cl-IB-MECA. Effect of Cl-IB-MECA (1 nM – 1 µM) on cell proliferation **(A, B)** and apoptosis **(C, D)** in IST-Mes2 **(A, C)** and MPP89 **(B, D)** cells. The Caspase 3/7 activation was used as the apoptosis parameter/marker. Results are represented as mean ± standard error of mean (SEM) of n=3 independent experimental replicates.

In the first phase of this study, *in vitro* experiments were carried out to investigate the effect of different concentrations of the A_3_AR agonist Cl-IB-MECA, from 1 nM to 1 µM, on cell proliferation and apoptosis. Assays were carried out in MPM cell lines IST-Mes2 and MPP89 (**Figures 2A–D**). Data showed that the highest effect of Cl-IB-MECA on cell proliferation inhibition and apoptosis induction was obtained at 100 nM (**Figures 2A–D**). For this reason, the 100 nM concentration of Cl-IB-MECA was chosen for the subsequent evaluations of the combined effect with the P2X7R antagonist AZ10606120 (300 nM).

### Impact of A_3_AR Agonist and P2X7R Antagonist on Cell Proliferation and Viability

**Figure 3:**
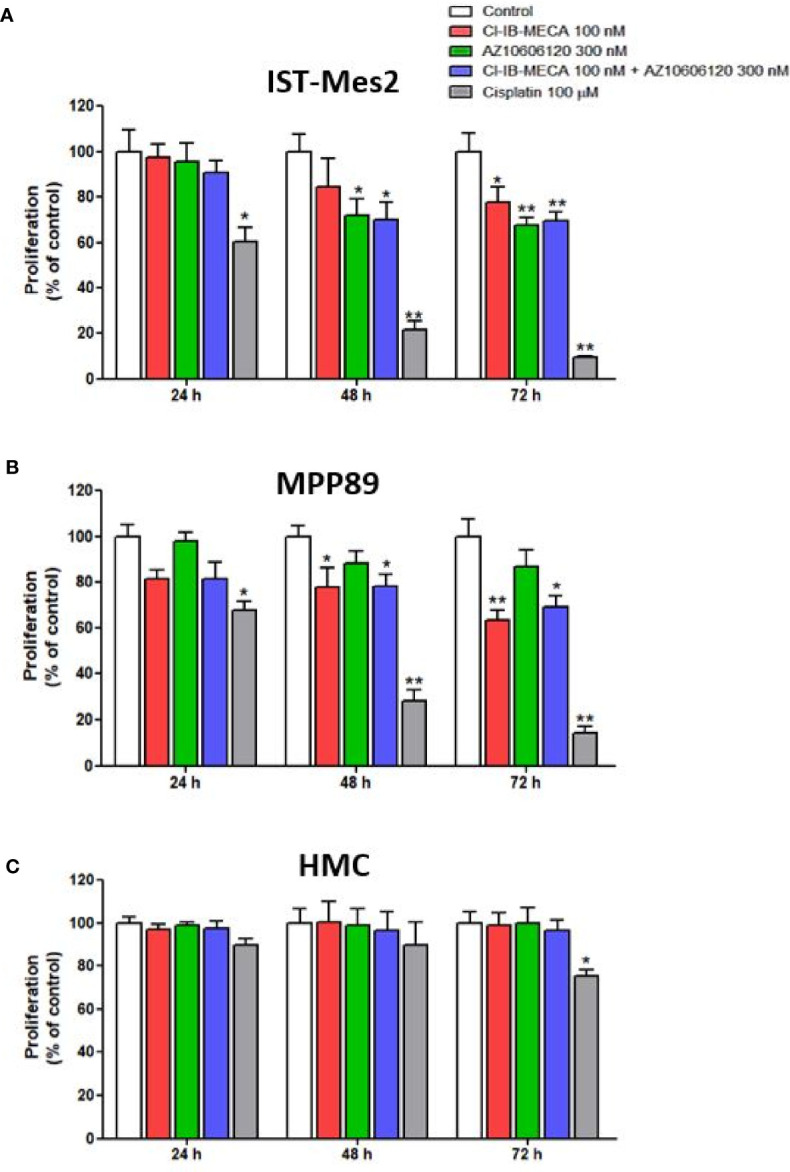
Impact of A_3_AR agonist and P2X7R antagonist on cell proliferation. Effect of Cl-IB-MECA (100 nM), AZ10606120 (300 nM), alone or in combination, in comparison to cisplatin 100 µM on cell proliferation in IST-Mes2 **(A)** and MPP89 **(B)** cells as well as in HMC cells **(C)**. Results are represented as mean ± standard error of mean (SEM) of n=3 independent experimental replicates. *p < 0.05 *vs* control; **p < 0.01 *vs* control.

In IST-Mes2 cells, a significant reduction of cell proliferation was obtained with Cl-IB-MECA at 72 hours of incubation (22%, p<0.05, **Figure 3A**). The P2X7R antagonist AZ10606120 inhibited IST-Mes2 proliferation after 48 hours (30%, p<0.05) and 72 hours (33%, p<0.01) of incubation (**Figure 2A**). The combination of Cl-IB-MECA and AZ10606120 inhibited IST-Mes2 proliferation at 48 and 72 hours, but the reduction did not significantly differ from the effect of the single compound (p>0.05, **Figure 3A**). Cisplatin significantly inhibited IST-Mes2 proliferation at all the time points investigated (p<0.05, **Figure 3A**).

In MPP89 cells, Cl-IB-MECA significantly reduced cell proliferation after 48 hours (22%, p<0.05) and 72 hours (37%, p<0.01) of treatment (**Figure 3B**). The AZ10606120 did not affect MPP89 cell proliferation at all the time points investigated (p>0.05, **Figure 3B**). As shown in **Figure 3B**, the combined effect of Cl-IB-MECA and AZ10606120 was not significantly different from the effect obtained treating the cells with Cl-IB-MECA alone (p>0.05, **Figure 3B**). Cisplatin, which was used as a positive control of the cell proliferation inhibitor, significantly inhibited MPP89 proliferation at all the time points investigated (p<0.01, **Figures 3A, B**).

Lastly, HMC proliferation was not affected by Cl-IB-MECA or AZ10606120, alone or in combination, at 24, 48 and 72 hours (p>0.05, **Figure 3C**). In addition, cisplatin was able to reduce HMC proliferation by 25% after 72 hours of incubation (p<0.05, **Figure 3C**).

**Figure 4:**
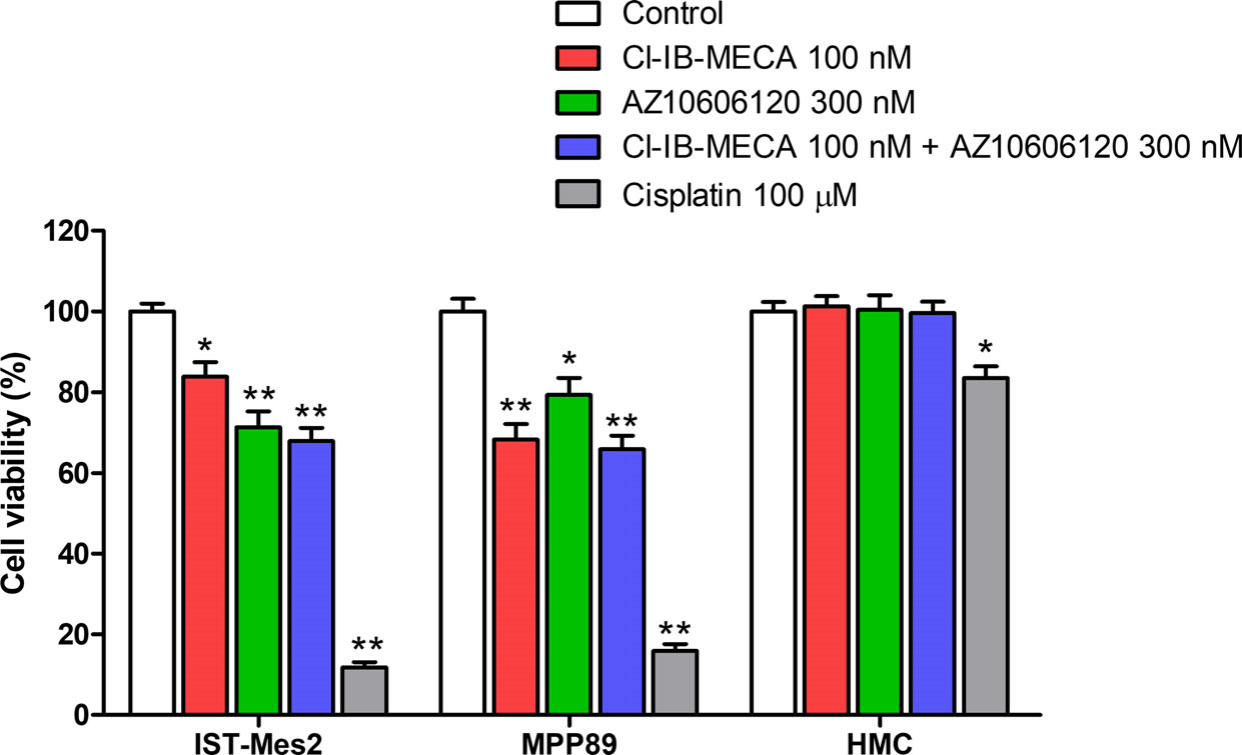
MTT assays. Effect of Cl-IB-MECA (100 nM), AZ10606120 (300 nM), alone or in combination, in comparison to cisplatin 100 µM on cell viability in IST-Mes2 and MPP89 cells as well as in HMC cells. Results are represented as mean ± standard error of mean (SEM) of n=3 independent experimental replicates. *p < 0.05 *vs* control; **p < 0.01 *vs* control.

Cell viability was evaluated by MTT assay after 72 hours of treatment with Cl-IB-MECA or AZ10606120, as single treatment and in combination, in comparison to cisplatin. In IST-Mes2 cells, a significant reduction of cell viability was obtained with 100 nM Cl-IB-MECA (16%, p<0.05, **Figure 4**). AZ10606120 decreased cell viability by 29% (p<0.01), an effect similar to that obtained with the concomitant treatment with the A_3_AR agonist and the P2X7 antagonist (32%, p<0.01, **Figure 4**). In MPP89 cells, a more evident effect of Cl-IB-MECA on cell viability reduction was observed (32%, p<0.01, **Figure 4**), while AZ10606120 decreased cell viability by 21% (p<0.05). The combined effect of Cl-IB-MECA and AZ10606120 (34%, p<0.01) was not significantly different from the effect obtained treating the cells with Cl-IB-MECA alone. HMC cell viability was not affected by Cl-IB-MECA or AZ10606120 treatment, alone or in combination (**Figure 4**). Cisplatin, used as positive control, significantly reduced cell viability in the three cell type investigated, with a more pronounced effect on MPM cell lines IST-Mes2 and MPP89 respect to HMC (**Figure 4**).

### Impact of A_3_AR Agonist and P2X7R Antagonist on Apoptosis

In the second phase of this study, the apoptotic effect of Cl-IB-MECA and AZ10606120, either as a single treatment or as a combination of the two drugs, was assayed in MPM cell lines. The caspase-3/7 activation, cleaved PARP-1 expression levels and cytochrome C release were used as the apoptosis parameters/markers. The etoposide and staurosporine drugs, which are well-documented apoptosis inducers, was used as a positive control (43).

**Figure 5:**
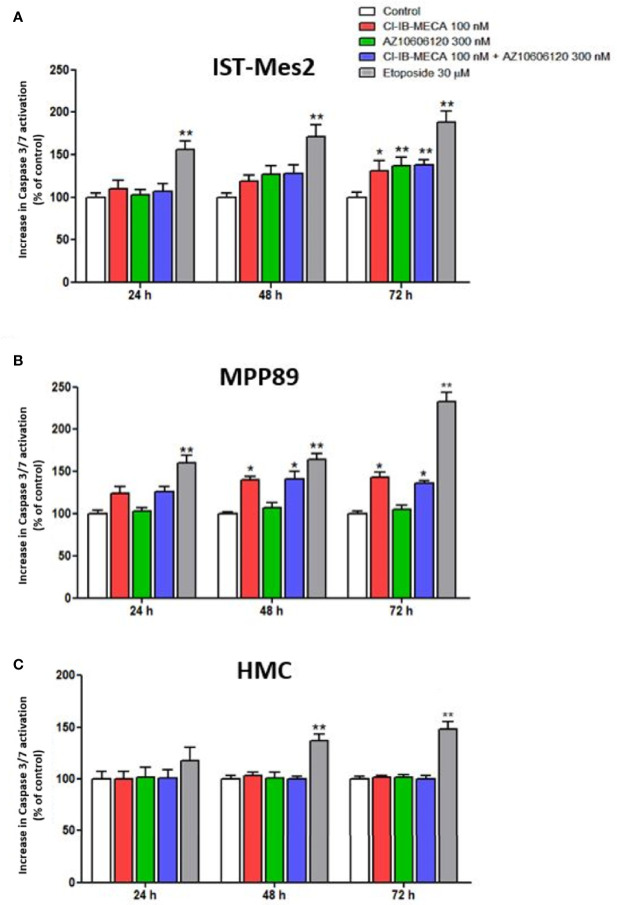
Impact of A_3_AR agonist and P2X7R antagonist on apoptosis. Effect of Cl-IB-MECA (100 nM), AZ10606120 (300 nM), alone or in combination, in comparison to etoposide 30 µM on cell apoptosis in IST-Mes2 **(A)** and MPP89 **(B)** cells as well as in HMC cells **(C)**. The Caspase 3/7 activation was used as the apoptosis parameter/marker. Results are represented as mean ± standard error of mean (SEM) of n=3 independent experimental replicates. *p < 0.05 *vs* control; **p < 0.01 *vs* control.

In IST-Mes2 cells, the 72 hours-treatment with Cl-IB-MECA induced a significant increase of caspase 3/7 activation (31%, p<0.05, **Figure 5A**). At the same time point, a more pronounced effect was obtained incubating the cells with AZ10606120 (37%, p<0.01). A similar effect was obtained treating IST-Mes2 cells with a combination of the two drugs (38%, p<0.01, **Figure 5A**). In MPP89, an induction of apoptosis was obtained by Cl-IB-MECA either at 48 hours (40%, p<0.05) and 72 hours (43%, p<0.05), whereas caspase 3/7 activation was not affected by AZ10606120 at all the time points investigated (p>0.05, **Figure 5B**). The combined treatment of MPP89 cells with Cl-IB-MECA and AZ10606120 did not enhance the effect of Cl-IB-MECA alone (p>0.05, **Figure 5B**). A significant caspase 3/7 activation was obtained with the positive control, etoposide, at all the time points investigated in IST-Mes2 and in MPP89 cells (p<0.01, **Figures 5A, B**). In HMC, neither Cl-IB-MECA nor AZ10606120 affected caspase 3/7 activation (p>0.05, **Figure 5C**). The positive control etoposide significantly induced HMC apoptosis after 48h and 72h of treatment (p<0.01, **Figure 5C**).

**Figure 6:**
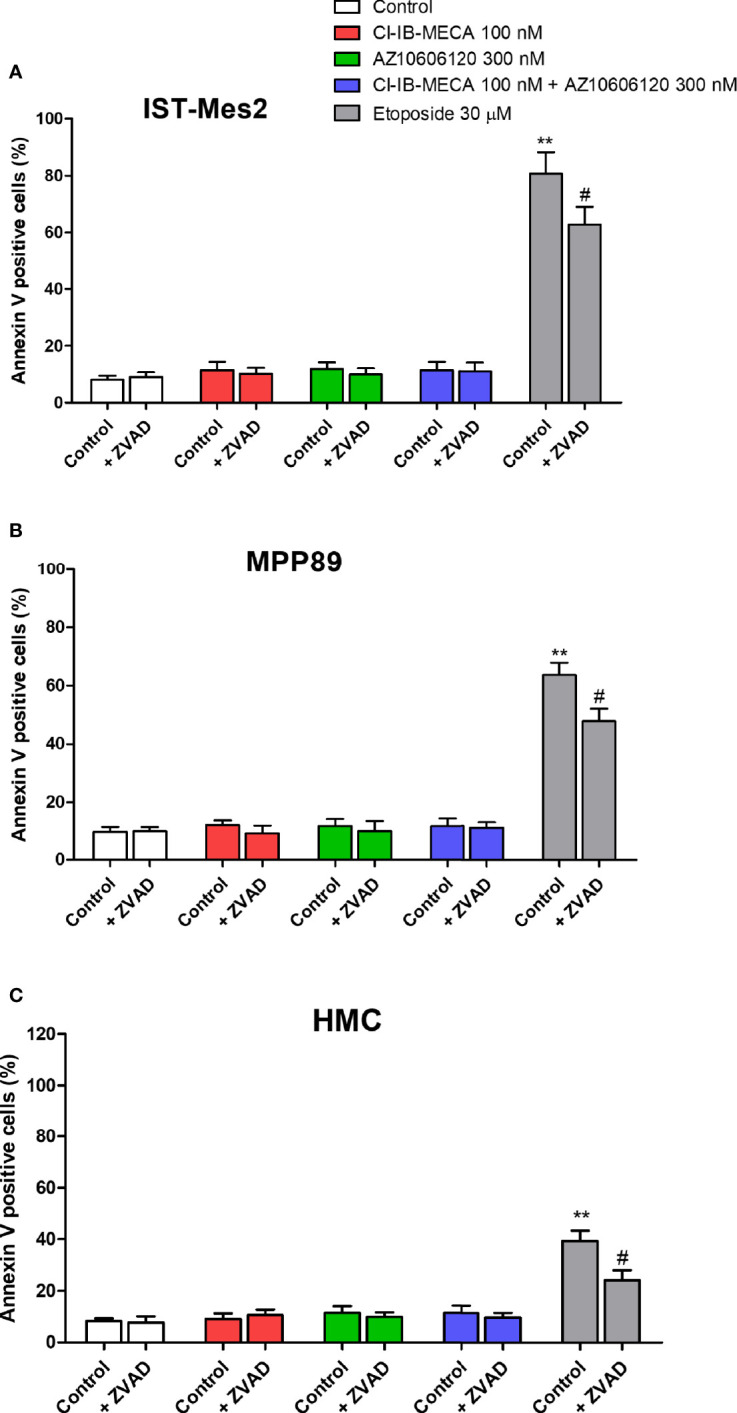
Annexin V assay. Effect of Cl-IB-MECA (100 nM), AZ10606120 (300 nM), alone or in combination, in comparison to etoposide 30 µM on the percentage of Annexin V positive cells in IST-Mes2 **(A)** and MPP89 **(B)** cells as well as in HMC cells **(C)**. Treatments were performed in the absence or in the presence of ZVAD-FMK (ZVAD, 50 µM) as a pan-caspase inhibitor. Results are represented as mean ± standard error of mean (SEM) of n=3 independent experimental replicates. **p < 0.01 *vs* control; ^#^p < 0.05 *vs* etoposide 30 µM.

As reported in **Figure 6**, 100 nM Cl-IB-MECA, 300 nM AZ10606120 or their combination for 72 hours were not able to increase the number of Annexin V positive cells in the MPM cell lines IST-Mes2 and MPP89 or in HMC cells. Etoposide at the 30 µM concentration significantly increased the percentage of Annexin V positive cells in all the cell type investigated. Treatment with ZVAD-FMK, a pan-caspase inhibitor, partially prevented etoposide effect (**Figure 6**).

**Figure 7:**
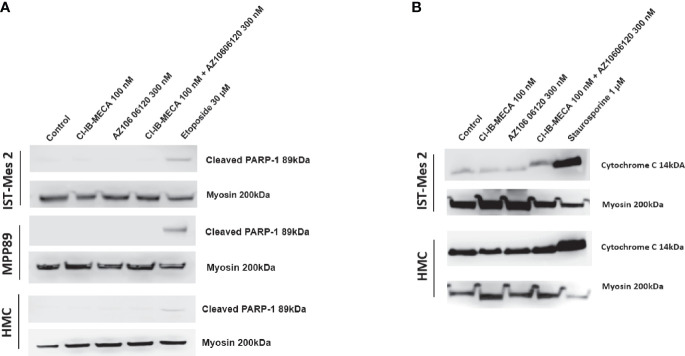
Western blot analysis of cleaved PARP-1 and cytochrome C. **(A)** The expression levels of cleaved PARP-1 have been determined in IST-Mes2 and MPP89 cells as well as in HMC cells after 72 hours of treatment with Cl-IB-MECA (100 nM), AZ10606120 (300 nM), alone or in combination, in comparison to etoposide 30 µM. **(B)** The cytochrome C release has been determined in the subcellular fraction of IST-Mes2 and HMC cells after 72 hours of treatment with Cl-IB-MECA (100 nM), AZ10606120 (300 nM), alone or in combination, in comparison to staurosporine 1 µM.

Lastly, cleaved PARP-1 expression levels were afterwards evaluated by western blot analysis in MPM cell lines IST-Mes2 and MPP89 as well as in HMC cells, while cytochrome C release was evaluated in the subcellular fraction of IST-Mes2 and HMC cells. No cleaved PARP-1 expression has been determined in IST-Mes2 and MPP89 cells treated with 100 nM Cl-IB-MECA, 300 nM AZ10606120, alone or in combination for 72 hours (**Figure 7A**). Similarly, a lack of cytochrome C release was determined in IST-Mes2 treated with 100 nM Cl-IB-MECA, 300 nM AZ10606120, alone or in combination for 72 hours (**Figure 7B**). As expected, etoposide and staurosporine, at the 30 µM and 1 µM concentration, respectively, increased the cleaved PARP-1 and cytochrome C levels in all the cell type investigated.

## Discussion

In this study, the A_3_AR agonist Cl-IB-MECA and the P2X7R antagonist AZ10606120 were investigated for their growth inhibition effects in MPM cell lines. Specifically, their activities as a single compound or in combination of the two drugs to verify their potential synergism were assayed *in vitro* on cell proliferation and apoptosis. The assays were carried out in MPM cell lines IST-Mes2 and MPP89 and in primary HMCs, employed as control. Single treatment with Cl-IB-MECA inhibited the cell proliferation and favored a pro-apoptotic effect in both IST-Mes2 and MPP89 MPM cell lines, whereas AZ10606120 showed the same anti-proliferative and pro-apoptotic effects in IST-Mes2, only. Cl-IB-MECA did not exhibit any synergistic effect with AZ10606120 in the MPM cell lines investigated.

Dose-response experiments (44) were initially carried out to investigate the effect of different concentrations of Cl-IB-MECA on cell proliferation/apoptosis in MPM cell lines. The highest anti-proliferative and pro-apoptotic effect of this compound was obtained at 100 nM. Thus, from these experiments, this concentration was chosen for the subsequent *in vitro* combination treatments. Results indicated that the inhibitory effect of Cl-IB-MECA on cell growth was evident in IST-Mes2 and MPP89 MPM cell lines, whereas, as expected, HMC did not show any growth inhibition. Furthermore, this A_3_AR agonist induced a significant pro-apoptotic effect in both MPM cell lines under investigation. Our data are in agreement with previous studies demonstrating that the *in vitro* stimulation of A_3_AR with Cl-IB-MECA induces anti-proliferative and pro-apoptotic effects in breast cancer (21), while inhibiting the metastatic progression in melanoma cells (22). The antitumor effect of Cl-IB-MECA has also been proven in mouse melanoma models (45).

Our results, together with previous data, confirm the role of A_3_AR activation with Cl-IBMECA in reducing the MPM cell growth. Accordingly, a previous study indicated that *in vitro* treatments of Cl-IB-MECA caused cytotoxicity, anti-proliferative and pro-apoptotic effects, through different molecular mechanisms, in both IST-Mes2 and MPP89 MPM cell lines (19). This antitumor capacity is linked to the Cl-IB-MECA-related stimulation of A_3_ARs, thereby decreasing proliferation and exerting a cytotoxic/pro-apoptotic effect *via* Akt/Nuclear Factor-kB signal transduction pathway (19). Indeed, the NF-kB pathway is involved in a number of cellular processes, such as proliferation, survival, and apoptosis (19). Since the inhibitory effect of Cl-IB-MECA on cell growth and apoptosis was evident in MPM cell lines, our results confirm the involvement of A_3_AR activation in inhibiting proliferation of MPM cells *in vitro*. For this reason, A_3_AR could be considered a pharmacological target to treat MPM affected patients.

It should be recalled that in contrast to the data reported in the studies mentioned above, two early investigations indicate that this A_3_AR agonist drug stimulated proliferation of both human colon cancer and astrocytoma cells (46, 47). Further studies are needed to clarify the role of A_3_AR in these tumors.

Currently, the potential role of A_3_AR agonist Cl-IB-MECA and P2X7R antagonist AZ10606120 on the MPM development/progression is unknown. For this reason, we investigated the anti-proliferative properties of Cl-IB-MECA in combination with AZ10606120 in MPM cell lines *in vitro*. Although without synergistic effect, our data demonstrate that Cl-IB-MECA, alone or in combination with AZ10606120, is very efficient in preventing MPM cell growth by inhibiting cell proliferation and inducing apoptosis. Similarly, single treatment of AZ10606120 reduced cell proliferation and apoptosis, but in IST-Mes2, only. Our data are in agreement with previous studies reporting that targeting P2X7R with its selective blocker AZ10606120 inhibited MPM cell growth on IST-Mes2 cell line, *in vitro* (34). The same study indicates that the systemic administration of the selective P2X7R blocker AZ10606120 inhibited *in vivo* growth of MPM tumors induced by MPM cells inoculated subcutaneously and/or intraperitoneally in nude mice (34). The antitumor proprieties of AZ10606120 have been reported in different cancers, such as neuroblastoma and pancreatic cancer (23, 24, 35). Indeed, the systemic administration of AZ10606120, as other P2X7R antagonists, reduced neuroblastoma growth in mice (35). The same P2X7R blocker showed inhibiting potential on cell proliferation, migration and invasion *in vitro* (23). In addition, it has been capable to reduce pancreatic stellate cells and collagen deposition in AZ10606120-treated mice (24). Our data, together with previous results, indicate that P2X7R might be a potential target for antitumor therapy, including MPM.

Although effective on cell proliferation inhibition and apoptosis, the combined treatment of Cl-IB-MECA and AZ10606120 did not have a synergistic effect. Despite the exact reason for the lack of synergism remains unclear, one possible reason might be the insufficient dosing of Cl-IB-MECA. Indeed, previous findings indicate that Cl-IB-MECA induce anti-proliferative effect at 0.01-10 mM in a dose dependent manner (20, 48). Herein, following dose-response experiments, the highest Cl-IB-MECA anti-proliferative/pro-apoptotic effect was determined at 100 nM. We therefore cannot exclude that a synergistic effect can be reached at Cl-IB-MECA concentrations above 100 nM. Further studies will clarify whether Cl-IB-MECA and AZ10606120 can be simultaneously used for anti-proliferative effect/antitumor therapy.

In conclusion, this is the first study reporting the *in vitro* effect of the A_3_AR agonist Cl-IB-MECA and the P2X7R inhibitor AZ10606120 in combination on cell proliferation and apoptosis in MPM cell lines. Single treatment with Cl-IB-MECA reduced cell growth and induced a pro-apoptotic effect in both MPP89 and IST-Mes2 MPM cell lines, whereas AZ10606120 was effective on cell proliferation inhibition and apoptosis in IST-Mes2, only. Although effective on cell proliferation inhibition and apoptosis in MPM cell lines, the combined treatment with Cl-IB-MECA and AZ10606120 did not exhibit a synergistic effect.

Further studies are needed to identify new combinations of antitumor drugs and more effective therapeutic strategies aimed at preventing MPM development.

## Data Availability Statement

The raw data supporting the conclusions of this article will be made available by the authors, without undue reservation.

## Author Contributions

FV, SP, and JCR carried out the experiments. FDV, KV, and MT contributed to conception and design of the study. FV, SP, and JCR performed the statistical analysis. KV and JCR wrote the first draft of the manuscript. FDV, KV, and MT edited the final version of the manuscript. All authors contributed to the article and approved the submitted version.

## Funding

FDV was supported by the Italian Association for Cancer Research (AIRC) grant n. IG 22883 and by the Ministry of University and Research of Italy, PRIN 2017 grant n. 8YTNWC. JCR was supported by the Italian Association for Cancer Research (AIRC) grant n. MFAG 21956. MT was supported by the Italian Association for Cancer Research (AIRC) grant n. IG 21617.

## Conflict of Interest

The authors declare that the research was conducted in the absence of any commercial or financial relationships that could be construed as a potential conflict of interest.

## Publisher’s Note

All claims expressed in this article are solely those of the authors and do not necessarily represent those of their affiliated organizations, or those of the publisher, the editors and the reviewers. Any product that may be evaluated in this article, or claim that may be made by its manufacturer, is not guaranteed or endorsed by the publisher.
